# Pessimistic cognitive biases mediate socioeconomic status and children’s mental health problems

**DOI:** 10.1038/s41598-023-32482-y

**Published:** 2023-03-30

**Authors:** Yu Hao, Gary W. Evans, Martha J. Farah

**Affiliations:** 1 Center for Neuroscience and Society, University of Pennsylvania, Philadelphia, PA USA; 2 Departments of Psychology and Human Centered Design, Cornell University, Ithaca, NY USA

**Keywords:** Human behaviour, Risk factors

## Abstract

Low socioeconomic status (SES) is associated with higher rates of emotional disorders in childhood and beyond. Here we assessed one possible contributor to this disparity, a cognitive bias in the interpretation of negative events, in a group of 341 9-year-olds (49% female, 94% White) ranging widely in SES. This cognitive bias, known as pessimism in the attributional style literature, is the tendency to interpret negative events as persistent (Stable) and pervasive (Global). It was found to be more common among lower SES children (effect sizes = 0.18–0.24 depending on SES measures: income to needs ratio, proportion of poverty from birth to age 9, and parental educational attainment). Moreover, persistent, pervasive adversity in children’s lives predicted this bias and mediated the SES—pessimism link. Pessimistic attributional style, in turn, was related to childhood emotional problems and mediated the relation between SES and these problems. Finally, evidence for serial mediation of the SES-mental health problems relationship was found via persistent, pervasive adversity and pessimism, respectively.

## Introduction

Pessimistic cognitive biases are associated with depression and other emotional disorders at all stages of life^1^. Such biases, in the theoretical framework of the Helplessness and Hopelessness theories of depression^2,3^ describe the ways in which people interpret negative events. The tendency to think of negative events as likely to persist *(Stability*) and as parts of a wider pattern in one’s life (*Globality*) defines the construct of pessimism. Early theorizing also included the tendency to attribute negative events to oneself rather than to circumstances (*Internality; the revised Helplessness theory of depression*^3^), but more recent formulations have identified Stability and Globality with the attributional style of pessimism and assigned Internality to a role in the construct of self-esteem (the Hopelessness theory of depression^2,4,5^).

Here we test a series of hypotheses about childhood socioeconomic status (SES) and the cognitive style of pessimism. Low SES has been associated with elevated levels of depression and other psychopathology throughout the lifespan^6^. Here we address three questions: First, we assess the relation between childhood SES and pessimistic attributional style. Second, we test a hypothesis concerning the underlying reasons for this relation. Third, we test the role of pessimistic attributional style in accounting for the SES-psychopathology relation in children.

Concerning the relation of attributional style to SES, little is known. Previous studies have assessed generally positive or negative expectations of life with items such as “In uncertain times, I usually expect the best” and “I rarely count on good things happening to me” from the Life Orientation Test^7–9^. Lower SES is typically associated with more negative expectations and an opposite trend for positive expectations^10–12^. These negative expectations are very naturally termed “pessimism,” but unlike the pessimistic attributional style of the Hopelessness Theory of Depression, they are an enduring trait, present in good times and bad, rather than interpretations attached to a negative event.

Only three studies have assessed SES and attributional style, all in adolescents, and these studies operationalized SES as a difference in degree of material and accompanying social deprivation in rural India. The first study, with adolescent boys, found that those suffering greater deprivation were more likely to attribute their failure on a task to internal (their ability or effort) rather than external (luck) factors^13^. A later study demonstrated that low SES adolescent boys showed more pessimistic attributional style for bad outcomes than low-deprived subjects, but no differences in attributing good outcomes using the scale Seligman and colleagues developed (Children’s Attributional Style Questionnaire^14^)^15^. Another study found that low SES adolescents (both genders) were more likely to attribute a single negative event (performing poorly on a task) to internal, stable, and global reasons^16^. In addition to focusing on one single event, this study used single-item probes to assess Internal, Stable, and Global attributions. Furthermore, all of these investigators did not examine the possible reason for the poverty-related attributional style and whether these poverty-related attributions might be linked to psychopathology, which are the second and third questions addressed by us here.

Therefore, we begin the present investigation with the relation of SES to pessimistic attributional style in childhood, operationalized SES measures including poverty and parental educational attainment. Then we proceed to test the second hypothesis, based on the relation of poverty to persistent, pervasive adversity. Pervious research on attributional style has suggested that pessimism may result from the adversity experienced by maltreated children^17^. The persistent, pervasive, adversity to which such children are exposed teaches them that negative events are likely to be stable and global features of the environment^17^. Poverty is also associated with negative events that are persistent and pervasive, from noise and crowding at home to separation from parents and witnessing violence^18–20^. Elevated exposure to threats and risk that are largely intractable may lead children growing up in low-income families to develop depressogenic attributional style. Given that low SES is also likely be associated with persistent pervasive adversity, it is possible that this feature of low SES mediates the relation between SES and pessimistic attributional style. We test this hypothesis with a mediation model. Last, we test the third hyopothesis of the role of attributional style in explaining mental health differences in children with varying SES background.

## Results

**Figure 1:**
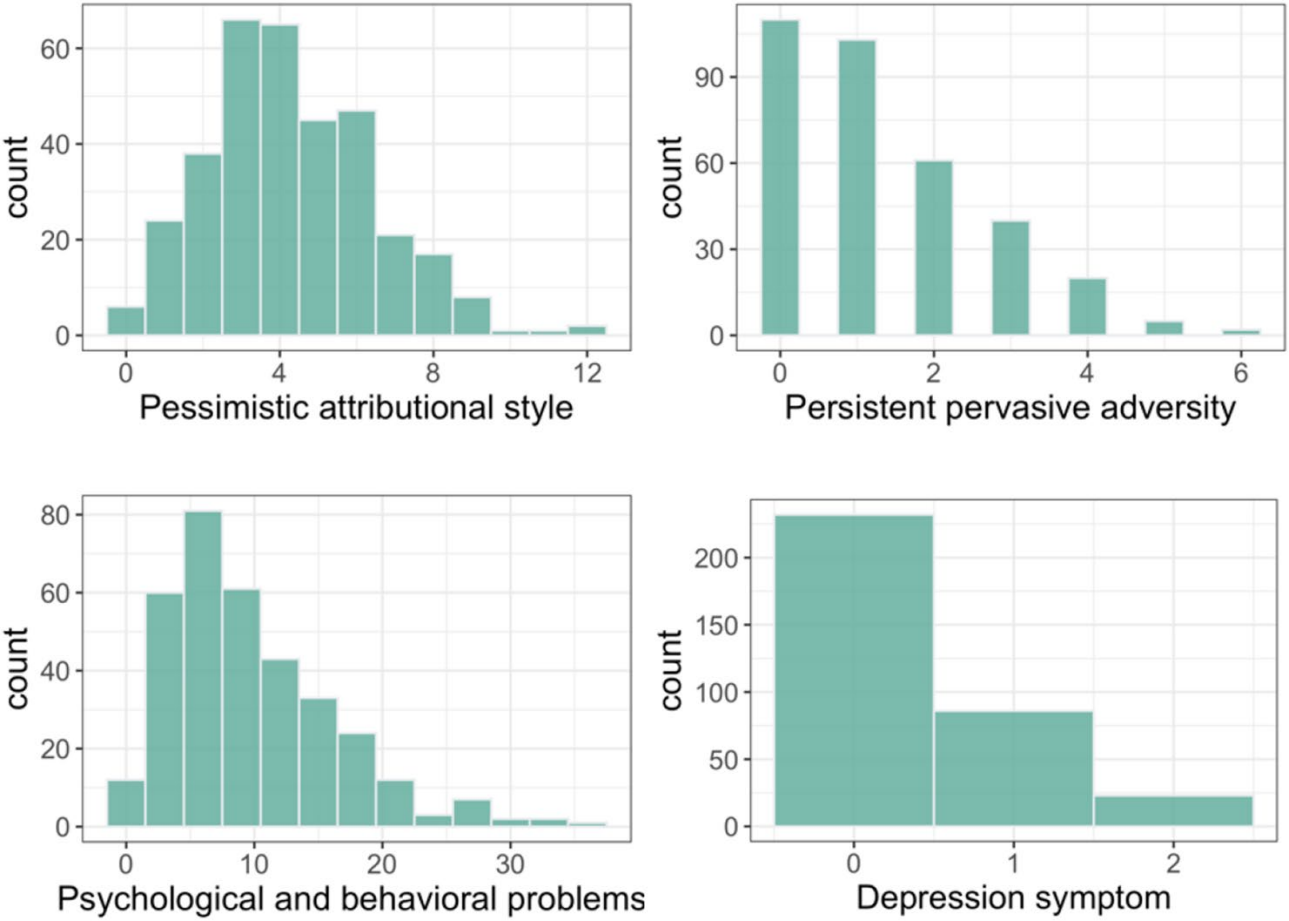
Distributions of pessimistic attributional style, persistent pervasive adversity, psychological and behavioral problems and the depression symptom from the Child Behavior Questionnaire.

The distributions of the psychological measures are shown in Fig. 1. Given the right skew of persistent, pervasive adversity and Child Behavior Questionnaire scores, they were square-root transformed before further analysis.

### SES and pessimistic attributional style

In answer to the first question, whether childhood SES is associated with pessimistic attributional style, we find that it is. As shown in Table 1, pessimism (the combination of Stability and Globality) is associated with all three measures of childhood SES.

**Table 1:** Relation of pessimistic attributional style to each measure of SES. Beta coefficients are standardized for ease of interpreting effect sizes and standard errors are in parentheses.

SES measures	Income to needs ratio age 9	Proportion of life in poverty	Parental education
Beta coefficient of SES relation to pessimism	− 0.22 (0.05) ***	0.24 (0.05) ***	− 0.18 (0.05) ***
*p*-value	*p *< 0.0001	*p *< 0.0001	*p *< 0.001
*Δ R*^*2*^	0.05	0.05	0.03

***p < 0.001; **p < 0.01; *p < 0.05.

Further investigation assessed the relation of SES to each of the three original scales of the Children’s Attributional Style Questionnaire^14^ individually: Stability and Globality (summed to make pessimism) as well as Internality (which is no longer viewed as relevant for measuring pessimism^5^). Stability and Globality were each significantly related to SES but Internality was not: for Stability and income to needs ratio (ITN), b = − 0.21, *p *< 0.001, *Δ R*^*2*^ = 0.04; for Globality and ITN, b = − 0.12, *p *= 0.022, *Δ R*^*2*^ = 0.02; and for Internality and ITN, b = 0.02, *p *= 0.730, *Δ R*^*2*^ = 0. Similar conclusions hold for the other two measures of SES, with *p*’s < 0.013 for the Stability and Globality, and *p *> 0.938 for Internality. None of the effects relating SES and pessimistic attributional style differ by gender.

### Developmental origins of pessimistic attributional style

Beginning with the relation of persistent, pervasive adversity to SES, we confirm previous findings that it is higher for lower SES children^18,21^. This is observed for all three measures of SES with absolute values of standardized beta coefficient range from 0.42 to 0.47 with all *p *< 0.0001, *Δ R*^*2*^ range from 0.17 to 0.22, depending on which SES measure.

We next test that persistent, pervasive adversity is associated with pessimism. We find support for this model, in that this pattern of adversity is associated with pessimistic attributional style: b = 0.27, S.E. = 0.05; *p *< 0.0001, *Δ R*^*2*^ = 0.07. Turning to the 3 separate scales of the Children’s Attributional Style Questionnaire, we find this relation for the two dimensions of pessimistic attributional style, Stability (b = 0.28, S.E. = 0.05; *p *< 0.0001, *Δ R*^*2*^ = 0.07) and Globality (b = 0.15, S.E. = 0.05; *p *= 0.006, *Δ R*^*2*^ = 0.02), but again do not find a relation with Internality (b = 0.01, S.E. = 0.05; *p *= 0.868, *Δ R*^*2*^ = 0). None of the effects relating SES to persistent pervasive adversity differ by gender.

**Figure 2:**
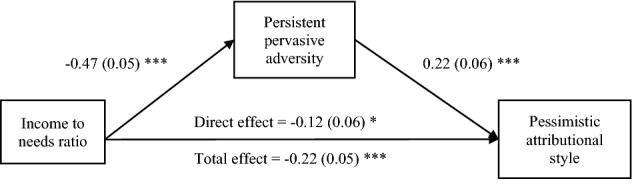
Path diagram showing mediation of the income to needs ratio and pessimistic attributional style relation by persistent pervasive adversity for children at age 9, controlling for gender. Standard coefficients on paths and standard errors in parentheses. As shown in Table 2, the indirect (mediated) path is statistically significant. ***p < 0.001; **p < 0.01; * *p* < 0.05.

Given the confirmed three pairwise relations among SES, pessimistic attributional style and persistent pervasive adversity, we went on to test whether or not the adversity measure accounts, statistically, for the association between SES and pessimistic attributional style. Here we see that the addition of persistent pervasive adversity to SES in the model predicting pessimistic attributional style reduces the SES-pessimism relation. Comparing the betas from Table 1 and Table 2, we see that the mediation account for 38–50% of the relation between SES and pessimism across all SES measures, showing significant mediations. We note that statistical mediation does not prove causation but is of interest because it is consistent with it, which it need not have been. Figure 2 depicts the diagram of mediation using ITN as an example and Table 2 shows the results for all three SES measures.

**Table 2:** Statistics of persistent pervasive adversity (PPA) mediating the SES-pessimism relation. Beta coefficients are standardized, and standard errors are in parentheses. Confidence intervals (95%) that do not incorporate 0 are bolded, indicating significant mediation.

	Income to needs ratio age 9	Proportion of life in poverty	Parental education
Direct path of SES predicting pessimism (with PPA in the model)	− 0.12 (0.06)*	0.15 (0.06)*	− 0.09 (0.06)
Indirect path of SES predicting pessimism through PPA SES PPA pessimism	− **0.157 to **− **0.049**	**0.041 to 0.141**	− **0.149 to **− **0.052**

***p < 0.001; **p < 0.01; *p < 0.05.

### Pessimism in relation to SES disparities in mental health

Attributional style is of interest primarily because of its role in mental health (although it has also been associated with academic achievement^22^). Consistent with such a role, we find that pessimistic attributional style is related to overall psychological and behavioral problems (b = 0.21, S.E. = 0.05; *p *< 0.0001, *Δ R*^*2*^ = 0.04). Although the strongest relations between pessimistic attributional style and mental health problems would be expected for depression, there was unfortunately only one depression-related item in the Child Behavior Questionnaire: “Often appears miserable, unhappy, tearful or distressed.” Despite the constraint on measurement sensitivity imposed by the use of just one item, pessimism does relate to depression (b = 0.25, S.E. = 0.11; *p *= 0.029). The pessimistic attributional relation to mental health does not differ by gender.

Before exploring the relevance of pessimistic attributional style to SES disparities in mental health, we first establish whether the present mental health questionnaire demonstrates the expected SES disparities. The expected relations are observed for all measures of SES: absolute values of standardized beta coefficient range from 0.31 to 0.38 with all *p *< 0.0001 (*Δ R*^*2*^ range from 0.10 to 0.14) depending on which SES measure. This relation is also significant for the single depression-related item: absolute values of the standardized beta coefficient range from 0.38 to 0.49 with *p *< 0.001. None of the effects relating SES and mental health differ by gender.

**Figure 3:**
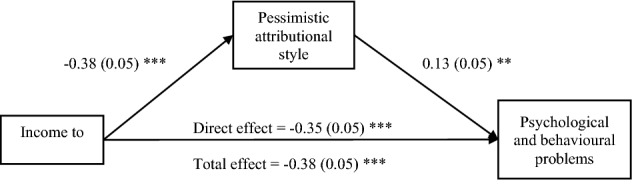
Path diagram showing mediation of the relation of income to needs ratio and psychological and behavioral problems by pessimistic attributional style for children at age 9, controlled for gender. Standard coefficients on paths and standard errors in parentheses. As shown in Table 3A, the indirect (mediated) path is statistically significant. ***p < 0.001; **p < 0.01; *p < 0.05.

The final hypothesis to be tested is that SES differences in mental health are mediated by pessimistic attributional style. As shown in Table 3A, pessimism mediates the SES-mental health relation across all SES measures, accounting for 6–10% of the effect (depending on the SES measure). Figure 3 depicts the diagram of mediation using ITN as an example. Also shown in Table 3B is the absence of significant mediation for the SES-depression relation. This null mediation result is difficult to interpret, given that depression was measured with just one item.

**Table 3:** Statistics of pessimistic attributional style mediating SES and psychological and behavioral problems (CBQ scale) (Table 3A) and depression symptom (Table 3B). Beta coefficients are standardized, and standard errors are in parentheses.

	Income to needs ratio age 9	Proportion of life in poverty	Parental education
(A)			
Total effect of SES predicting CBQ	− 0.38 (0.05) ***	0.31 (0.05) ***	− 0.36 (0.05) ***
Direct path of SES predicting CBQ (with pessimism in the model)	− 0.35 (0.05) ***	0.28 (0.05) ***	− 0.34 (0.05) ***
Indirect path of SES prediting CBQ through pessimsm SES pessimism CBQ	− **0.061 to **− **0.007**	**0.008 to 0.068**	− **0.055 to **− **0.006**
(B)			
Total effect of SES predicting depressive symptom	− 0.38 (0.13) **	0.40 (0.12) **	− 0.49 (0.13) ***
Direct path of SES predicting depressive symptom (with Pessimism in the model)	− 0.34 (0.13) **	0.36 (0.12) **	− 0.46 (0.13) ***
Indirect path of SES predicting depressive symptom through pessimsm: SES pessimism depression symptom	− 0.034 to 0.011	− 0.041 to 0.013	− 0.034 to 0.010

*** *p* < 0.001; ** *p* < 0.01; * *p* < 0.05.

Confidence intervals (95%) that do not incorporate 0 are bolded, indicating significant mediation.

**Figure 4:**
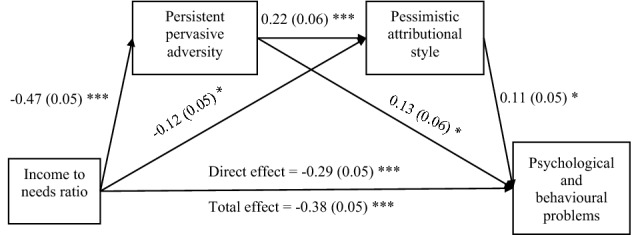
Path diagram showing mediation of the relation of income to needs ratio and psychological and behavioral problems by persistent pervasive adversity and pessimistic attributional style for children at age 9, controlled for gender. As shown in Table 4 the indirect (mediated) path is statistically significant. Standard coefficients on paths and standard errors in parentheses. ***p < 0.001; **p < 0.01; *p < 0.05.

The foregoing analyses addressed the three central questions of this study, and all three were answered in the affirmative: We found that SES is related to pessimistic attributional style, that persistent, pervasive adversity at least partly mediates that relation, and that pessimistic attributional style at least partly mediates the relation between SES and psychological and behavioral problems. Having confirmed the hypothesized inter-relations of the four variables when considered two or three at a time, we now report one final analysis of all paths involving all variables together. Figure 4 shows the path analysis diagram with income to needs ratio as an example. Results of all SES measures are shown in Table 4 which shows the significance of all indirect paths, including the serial mediation of the SES-psychological and behavioral problems relation by persistant pervasive adversity and pessimistic attributional style. The results highlight the influence of persistent, pervasive adversity on its own and through pessimistic attributional style.

**Table 4:** Statistics of persistent pervasive adversity (PPA) and pessimistic attributional style mediating SES and psychological and behavioral problems (CBQ scale). Confidence intervals (95%) that do not incorporate 0 are bolded, indicating significant mediation.

	Income to needs ratio age 9	Proportion of life in poverty	Parental education
Indirect path: SES --> PPA --> pessimism --> CBQ	− **0.025 to **− **0.001**	**0.001 to 0.023**	− **0.025 to **− **0.001**
Indirect path: SES --> PPA --> CBQ	− **0.118 to **− **0.010**	**0.027 to 0.132**	− **0.113 to **− **0.019**
Indirect path: SES --> pessimism --> CBQ	− 0.037 to 0.001	**0.000 to 0.042 #**	− 0.031 to 0.004

^#^0 because round to three decimal places, real number greater than 0.

## Discussion

Here we report that children from lower SES families tend to have a more pessimistic attributional style. That is, they are biased to understand negative events as Stable (persistent) and Global (pervasive) aspects of their lives. We also found evidence suggesting why this might be: children in poverty experience persistent and pervasive adversity and therefore come to expect negative events that occur will be persistent and pervasive (cf. Rose & Abramson^17^). Further, we found the resulting pessimistic attributions to be predictive of low SES children’s susceptibility to psychological disorders.

The current study has a number of limitations. Two concern the available measures. Depression was measured by a single item on a parent questionnaire, which is disappointing since the literature on attributional style and mental health is mostly focused on depression. We nevertheless found our limited depression measure to be related to the pessimistic attributional style as well as to SES. Another limitation was the use of an early measure of attributional style, the Child Attributional Style Questionnaire, which predates revised theories of attributional style and more recently designed and validated questionnaires for children (e.g., Children’s Cognitive Style Questionnaire^23^). Assessing attributional style more directly, following actual versus hypothetical negative life events, would increase study validity. The single time-point of measurement at a single age also limits the confidence with which we can infer causal relations, either between adversity and pessimism or between pessimism and psychopathology. Finally, the majority of the children in our sample were White and rural, so we cannot generalize to other samples or settings.

Within the limits imposed by these methodological shortcomings, the present findings provide important clues for understanding and preventing the elevated risk of behavioral and psychological problems in low SES children. Given that pessimistic attributional style is a source of vulnerability to psychopathology, the greater pessimism of lower SES children may explain the greater incidence of psychopathology among such children. This inference is bolstered by the mediation analyses presented here, advancing our understanding of SES disparities in mental health.

Concerning prevention, the role of biased cognition in the childhood emotional disorders has suggested to some that teaching healthier patterns of cognition could help prevent psychopathology in children. Unfortunately, previous intervention efforts aimed at changing children’s attributional style have proven only minimally helpful^24^. Although more effective cognitive interventions may be possible, the present findings suggest a reason why targeting cognition in childhood adversity may not yield the hoped-for results. The pessimistic cognitive bias, at least as it relates to SES, is rooted in lived experience and may not be changeable by explicit teaching while the same life conditions persist. It is possible that reducing pessimism and emotional disorders will require intervening on root causes, the persistence and pervasiveness of negative events in childhood poverty. While much harder to accomplish than instruction on explicit cognitive attributions, it appears to be effective; interventions to increase material resources have led to improvements in emotional wellbeing^25–27^.

## Methods

### Participants

Participants (*N* = 341, *Age M* = 9.18 years old, *SD* = 1.17, 49% female, 94% Caucasian, 3% African American, all other ethnicities less than 3%) were recruited through public schools, Co-Operative Extension, and various antipoverty programs in the rural northeast of the United States^28^. The experimental protocols were approved by Cornell University Institutional Review Boards. All methods were performed in accordance with the relevant guidelines and regulations. Informed consent was obtained from all participants and/or their legal guardians. All the questionnaires used in the current study were administered during the home visit and all participants were from different families.

### Measures

#### Socioeconomic status

**Figure 5:**
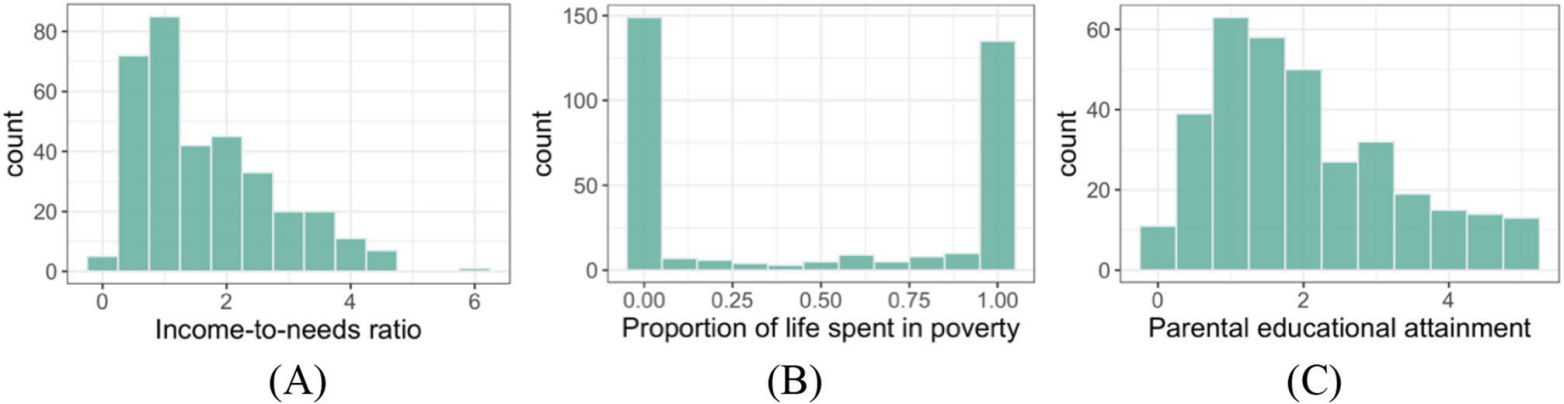
Distributions of each SES measure.

SES was measured in three ways. Two of the SES measures were collected at age 9 which was the same as mental health and attributional style measures, whereas the other SES measure was collected retrospectively between birth and age 9. The first was based on the family’s income-to-needs (ITN) ratio, that is, total family income divided by the number of people in the family. The poverty threshold is an ITN ratio determined by the US government based on the consumer price index. Our main income measure was the family’s ITN expressed as the factor by which family ITN exceeded or fell short of the federal poverty threshold, with an ITN of 1.0 defining the poverty threshold. Another income-based measure was the proportion of life spent in poverty from birth to age 9, determined from income and needs levels calculated every six months. Finally, parental educational attainment was a third measure of the child’s SES. We averaged the level of maternal and paternal educational attainment. Education was coded 0 to 5, each corresponding to 0 = less than high school, 1 = high school graduate, 2 = some college, 3 = 2 year college degree, 4 = 4 year college degree, 5 = graduate work. Figure 5 shows the distributions of these SES measures. In sum, the sample tended to be low-income, and most parents did not have a two- or four-year college degree.

#### Children’s attributional style

Children’s Attributional Style Questionnaire^14^ was administered to children to assess attributional style for negative events. This questionnaire was formulated before Internality was eliminated as a component of pessimism, so data on Internality was also collected. We therefore report the results from the Internality measure, but use the results from Stability and Globality to measure pessimistic attributional style^5^. Examples of each included “You fail a test. Choose the alternative closest to the way you’d really feel if that particular thing happened to you.” Choices are ‘My teacher makes hard tests’ or ‘The past few weeks, my teacher has made hard tests’ [Stability]. “A person steals money from you.” Choices are ‘People are dishonest’ or ‘That person is dishonest’ [Global]. “You get a bad grade in school.” Choices are ‘I am stupid’ or ‘Teachers are unfair graders’ [Internal]. Choices were coded 0 or 1, with 1 indicating a pessimistic attributional choice. Each attributional style category comprised 8 items, resulting in a score from 0 to 8 for each category and 0 to 16 for the composite of Stability and Globality used to measure pessimistic attributional style. The Cronbach alpha value for the Pessimism is 0.52 and for the Internality is 0.39. Low reliability would attenuate any relations found in our study.

#### Persistent pervasive adversity

We measured persistent pervasive adversity, of the kind hypothesized by Rose and Abrahms^17^ to cause pessimistic attributional styles in abused children, by assessing physical and psychosocial adversities from an index of cumulative risk^28,29^. Home visits were used to assess three physical adversities linked to the home environment: crowding, noise and housing quality. Crowding was defined as persons/room; noise by two, two-hour decibel readings in the home; and housing quality by a standardized rating instrument conducted during a walk through of the residence^30^. Maternal report elicited by the Life Events and Circumstances checklist^31,32^ was used to assess three psychosocial adversities: violence, family turmoil, and child separation from parents.

#### Psychological and behavioral problems

The Child Behavior Questionnaire^33^ was rated by parents to assess a range of child behaviors that are potentially indicative of psychological problems. It consists of 17 statements about child behavior that the parent rates as 0 = doesn’t apply, 1 = applies somewhat, and 2 = certainly applies. It is not intended to differentiate among different types of psychological problems, but instead gives an overall level of dysfunction. The Cronbach alpha value for the CBQ is 0.82, indicating good reliability. We used total score of CBQ to index mental health measure.

There was just one statement that was clearly depression related. Given the special relevance of attributional style to depression, we also analyze this one separately: “Often appears miserable, unhappy, tearful or distressed.”

### Data analyses

Data were missing completely at random, so multiple imputation of missing data was performed^29^. We first examined the distributions of all psychological measures to evaluate necessary transformations. All coefficients reported will be standardized to make effect sizes comparable across measures.

We then assessed the strength of the relation between SES and the pessimistic attributional style using a multiple linear regression model. This is the primary prediction concerning the SES and the results of the Children’s Attributional Style Questionnaire. For this and subsequent analyses, we carried out separate anslyses for each measure of SES. We also covaried gender and an SES-gender interaction term, in order to check the equivalence of the SES effect in boys and girls. The interaction term is dropped from models when nonsignificant.

In a further examination of specific attributional styles, we analyzed the relation of SES to the three original subtypes of the attributional style, Stability, Globality and Internality, each measured as a score out of 8. This was carried out within a single mixed effect model, with attribution subtype as a categorical factor and participant as a random effect. The interaction of SES and three attribution subtypes provided a measure of the differing strength of the relationships between SES and the these subtypes. Mixed effect modeling was carried out with the package “lme4” (https://github.com/lme4/lme4/) in R. Post-hoc analysis to reveal SES relations to each attributional subtype, and to compute the marginal means of linear relations and pairwise compared these relations correcting multiple comparisons, was accomplished using the “emmeans” (Estimated Marginal Means) package in R^34^.

If SES is indeed found to be related to degree of pessimistic attributional style, then we will turn to the question of how this comes about. We test the role of persistent, pervasive adversity, suggested by the theorizing of Rose and Abramson^17^, as a candidate factor responsible for the relation of pessimistic attributional style and SES. To do so, we would first assess the relation between persistent, pervasive adversity and SES, using multiple regression as before. If higher levels of persistent, pervasive adversity are associated with lower SES, then a mediation analysis would be carried out to see whether persistent, pervasive adversity statistically mediates the SES-pessimism relation. The standardized indirect pathway linking SES and pessimistic attributional style through persistent, pervasive adversity (the mediated pathway) will be estimated with 95% bias-corrected confidence intervals based on bootstrapping with 50,000 samples with the PROCESS macro in SPSS^35^.

With the same mediation analysis approach, we also examined if attributional style could mediate the relation of SES to children’s psychological problems in general and depression in particular.

## Data Availability

The datasets generated during and/or analysed during the current study are available from the corresponding author on reasonable request.
